# The Effect of Sex on Disease Stage and Survival after Radical Cystectomy in Non-Urothelial Variant-Histology Bladder Cancer

**DOI:** 10.3390/jcm12051776

**Published:** 2023-02-23

**Authors:** Rocco Simone Flammia, Antonio Tufano, Francesco Chierigo, Christoph Würnschimmel, Benedikt Hoeh, Gabriele Sorce, Zhen Tian, Umberto Anceschi, Costantino Leonardo, Francesco Del Giudice, Carlo Terrone, Antonio Giordano, Andrea Morrione, Fred Saad, Shahrokh F. Shariat, Alberto Briganti, Francesco Montorsi, Felix K. H. Chun, Michele Gallucci, Pierre I. Karakiewicz

**Affiliations:** 1 Department of Maternal-Child and Urological Sciences, Policlinico Umberto I Hospital, Sapienza University of Rome, 00161 Rome, Italy; 2 Cancer Prognostics and Health Outcomes Unit, Division of Urology, University of Montréal Health Center, Montréal, QC H4A 3J1, Canada; 3 Sbarro Institute for Cancer Research and Molecular Medicine, Center for Biotechnology, Department of Biology, College of Science and Technology, Temple University, Philadelphia, PA 19122, USA; 4 Department of Surgical and Diagnostic Integrated Sciences (DISC), University of Genova, 16146 Genova, Italy; 5 Martini-Klinik Prostate Cancer Center, University Hospital Hamburg-Eppendorf, 20251 Hamburg, Germany; 6 Department of Urology, University Hospital Frankfurt, Goethe University Frankfurt am Main, 60596 Frankfurt am Main, Germany; 7 Division of Experimental Oncology/Unit of Urology, URI, Urological Research Institute, IRCCS San Raffaele Scientific Institute, 20132 Milan, Italy; 8 Department of Urology, Regina Elena National Cancer Institute, 00144 Rome, Italy; 9 Department of Medical Biotechnology, University of Siena, 53100 Siena, Italy; 10 Department of Urology, Weill Cornell Medical College, New York, NY 10065, USA; 11 Department of Urology, University of Texas Southwestern, Dallas, TX 75390, USA; 12 Department of Urology, Second Faculty of Medicine, Charles University, 128 08 Prague, Czech Republic; 13 Institute for Urology and Reproductive Health, I.M. Sechenov First Moscow State Medical University, 119991 Moscow, Russia; 14 Hourani Center for Applied Scientific Research, Al-Ahliyya Amman University, Amman 11942, Jordan; 15 Department of Urology, Comprehensive Cancer Center, Medical University of Vienna, 1090 Vienna, Austria

**Keywords:** muscle-invasive bladder cancer, adenocarcinoma, neuroendocrine carcinoma, variant-histology, squamous cell carcinoma, radical cystectomy

## Abstract

Background: Female sex in patients treated by radical cystectomy (RC) is associated with more advanced stage and worse survival. However, studies supporting these findings mostly or exclusively relied on urothelial carcinoma of the urinary bladder (UCUB) and did not address non-urothelial variant-histology bladder cancer (VH BCa). We hypothesized that female sex is associated with a more advanced stage and worse survival in VH BCa, similarly to that of UCUB. Materials and Methods: Within the SEER database (2004–2016), we identified patients aged ≥18 years, with histologically confirmed VH BCa, and treated with comprehensive RC. Logistic regression addressing the non-organ-confined (NOC) stage, as well as cumulative incidence plots and competing risks regression addressing CSM for females vs. males, were fitted. All analyses were repeated in stage-specific and VH-specific subgroups. Results: Overall, 1623 VH BCa patients treated with RC were identified. Of those, 38% were female. Adenocarcinoma (*n* = 331, 33%), neuroendocrine tumor (*n* = 304, 18%), and other VH (*n* = 317, 37%) were less frequent in females but not squamous cell carcinoma (*n* = 671, 51%). Across all VH subgroups, female patients had higher NOC rates than males did (68 vs. 58%, *p* < 0.001), and female sex was an independent predictor of NOC VH BCa (OR = 1.55, *p* = 0.0001). Overall, five-year cancer-specific mortality (CSM) were 43% for females vs. 34% for males (HR = 1.25, *p* = 0.02). Conclusion: In VH BC patients treated with comprehensive RC, female sex is associated with a more advanced stage. Independently of stage, female sex also predisposes to higher CSM.

## 1. Introduction

Sex has shown to be an important predictor of survival in urothelial carcinoma of the urinary bladder (UCUB) [1,2,3]. Specifically, radical cystectomy (RC) in female patients is associated with a more advanced stage and worse survival as compared with males [4,5,6,7,8]. However, studies supporting these findings mostly or exclusively relied on UCUB and did not address non-urothelial variant-histology bladder cancer (VH BCa), as reported in a recent systematic review and metanalysis by Uhlig et al. (59 studies, *n* = 69,666) [9]. Consequently, the association between sex and either advanced stage or worse survival in VH Bca treated with RC is unknown. To address this void, we relied on SEER database and examined four VH Bca subgroups: patients with squamous cell carcinoma (SCC), adenocarcinoma (ADK), neuroendocrine carcinoma (NE) and other types (other VH), according to the 2016 World Health Organization (WHO) classification [10,11]. Mixed histology is not coded in the SEER database; thus, these criteria reflect the predominant histologic subtype [12]. We hypothesized that female sex is associated with a more advanced stage and worse survival in VH BCa, similar to that of UCUB.

## 2. Materials and Methods

### 2.1. Study Population

Within the SEER database (2004–2016), we identified radical cystectomy (RC) patients, aged ≥ 18 years old, with a histologically confirmed diagnosis of BCa (International Classification of Disease for Oncology site code C67.0-9), and no distant metastasis according to the American Joint Committee on Cancer (AJCC), Seventh Edition. Only patients harboring VH BCa, consisting of either SCC, ADK, NE, and other types (other VH), were included. Moreover, in accord with previous methodology [8], only patients with comprehensive RC that included lymphadenectomy were selected [13]. Patients with disease confirmed by autopsy, death certificate-only cases, and patients exposed to radiotherapy were excluded.

### 2.2. Statistical Analysis

The analysis consisted of two main parts. First, we examined the association between female sex and non-organ-confined stage (NOC = T3-4 and/or N1-3) in univariable and multivariable logistic regression models (LRM) after adjustments for age. All analyses were then repeated in each of four VH-specific subgroups: SCC, ADK, NE, and other VH.

Second, we focused on cancer-specific mortality (CSM) according to female sex and relied on cumulative incidence plots and competing risks regression (CRR) models. Covariates consisted of age, T-stage, N-stage, chemotherapy (CHT), and further adjustment for other-cause mortality (OCM) was performed. All analyses were repeated after stratification according to stage-specific (NOC vs. organ-confined [OC]), as well as VH-specific subgroups (SCC, ADK, NE, other VH). All tests were two-sided with a level of significance set at *p* < 0.05 and R software for statistical computing and graphics (version 3.4.3) was used for all analyses.

## 3. Results

### 3.1. Descriptive Characteristics of Study Population

Overall, 1623 patients with VH BCa treated with RC were identified (Table 1). Of those, 38% were female. ADK was less frequent in females than in males (*n* = 331, 33%), NE (*n* = 304, 18%), and other VH (*n* = 317, 37%), but SCC was not (*n* = 671, 51%). No difference in median age was recorded between females vs. males (67 vs. 68 years).

### 3.2. The Association of Sex with Non-Organ-Confined (NOC) VH BCa

**Figure 1:**
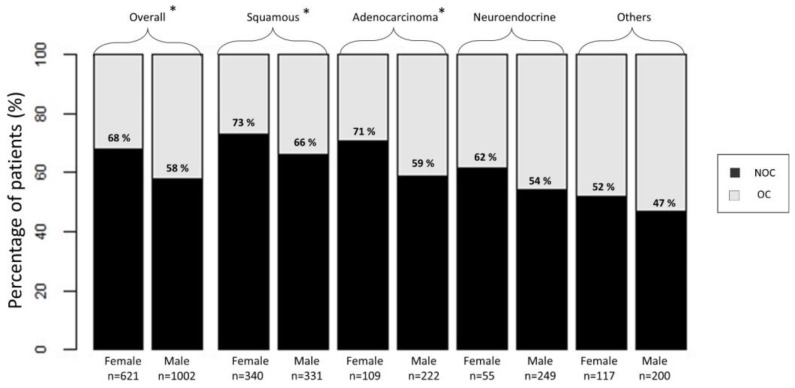
Stacked bar plots depicting stage at presentation according to patient sex in the overall cohort of non-urothelial variant-histology bladder cancer (VH BCa) treated with radical cystectomy and within each VH-specific subgroup. Stages were defined as non-organ-confined (NOC) vs. organ-confined (OC). * *p* < 0.05.

Across all VH subtypes (Figure 1), female patients had higher NOC rates than males (68 vs. 58%, *p* < 0.001), and female sex was an independent predictor of NOC VH BCa (OR = 1.55, 95% CI 1.26−1.92, *p* < 0.001).

After stratification according to VH-specific subgroups (Figure 1), female patients exhibited higher NOC rates in SCC (73% vs. 66%, *p* = 0.046) and ADK (71% vs. 59%, *p* = 0.040). In SCC and ADK subgroups, female sex was also an independent predictor of NOC VH BCa (Table 2). Conversely, no differences in NOC rates between females and males were detected in NE (62% vs. 54%, *p* = 0.3) and other VH (52% vs. 47%, *p* = 0.4). In NE and other VH, female sex was also not an independent predictor of NOC VH BCa (Table 2).

### 3.3. Effect of Female Sex in Cancer-Specific Mortality (CSM)

#### 3.3.1. CSM in the Overall Cohort

**Figure 2:**
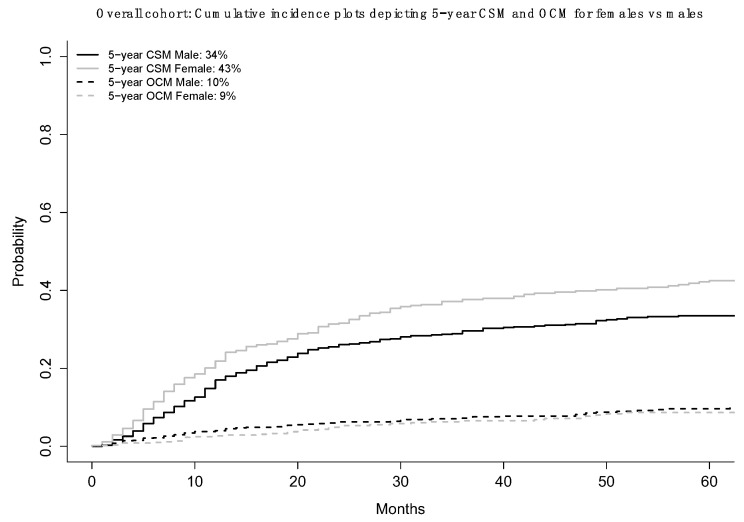
Cumulative incidence plots depicting 5-year cancer-specific mortality (CSM) and 5-year other-cause mortality (OCM) in the overall cohort of non-urothelial variant-histology bladder cancer (VH BCa) according to sex (female vs. male).

Across all VH subtypes (*n* = 1623), five-year CSM rates were 43% for females vs. 34% for males (Figure 2). This translated into a CRR HR of 1.25 (95% CI 1.04–1.50, *p* = 0.02) after adjustment for age, T-stage, N-stage, VH, CHT, and OCM (Table 2).

#### 3.3.2. CSM according to Stage-Specific Subgroups (OC vs. NOC)

**Figure 3:**
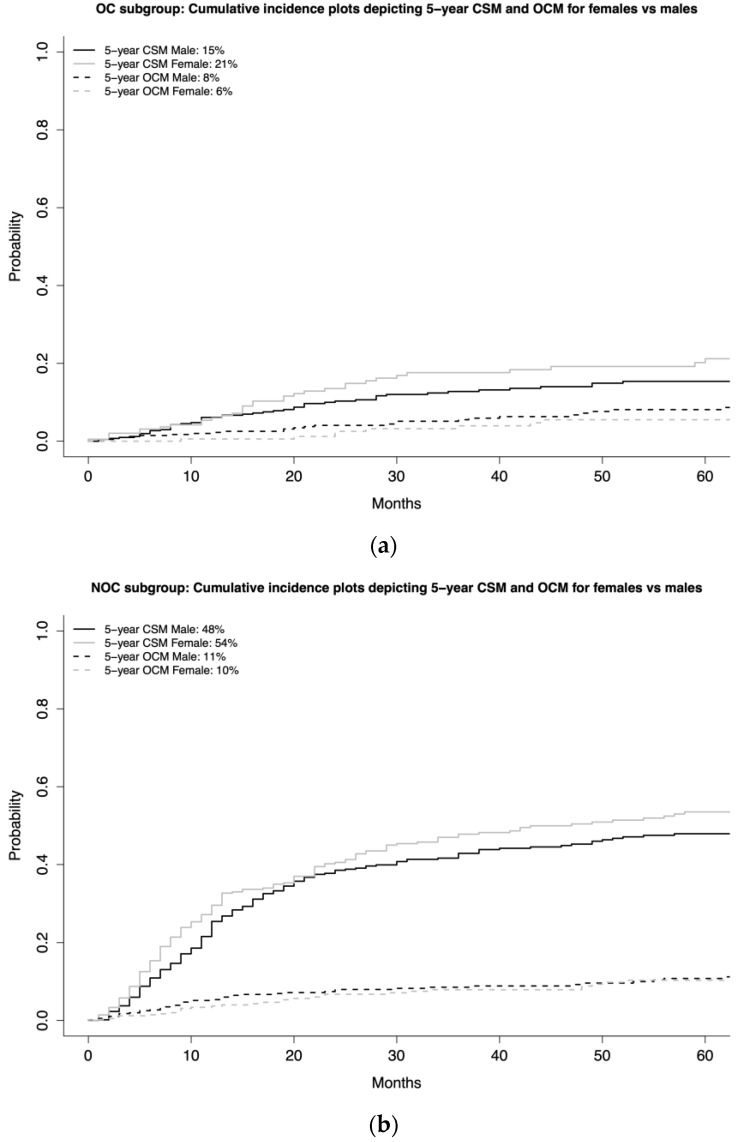
Cumulative incidence plots depicting 5-year cancer-specific mortality (CSM) and 5-year other-cause mortality (OCM) according to sex (female vs. male) in both the organ-confined (OC) (**a**) as well as non-organ-confined (NOC) subgroup (**b**).

In the OC subgroup (*n* = 623), five-year CSM rates were 21% for females vs. 15 % for males (Figure 3a). This translated into a CRR HR of 1.65 (95% CI 1.08–2.52, *p* = 0.02) after adjustment for age, T-stage, VH, CHT, and OCM (Table 2). In the NOC subgroup (*n* = 1000), five-year CSM rates were 54% for females vs. 48% for males (Figure 3b). This translated into a CRR HR of 1.17 (95% CI 0.96–1.43, *p* = 0.1) after adjustment for age, T-stage, N-stage, VH, CHT, and OCM (Table 2).

#### 3.3.3. CSM according to VH-Specific Subgroups (SCC, ADK, NE, other VH)

**Figure 4:**
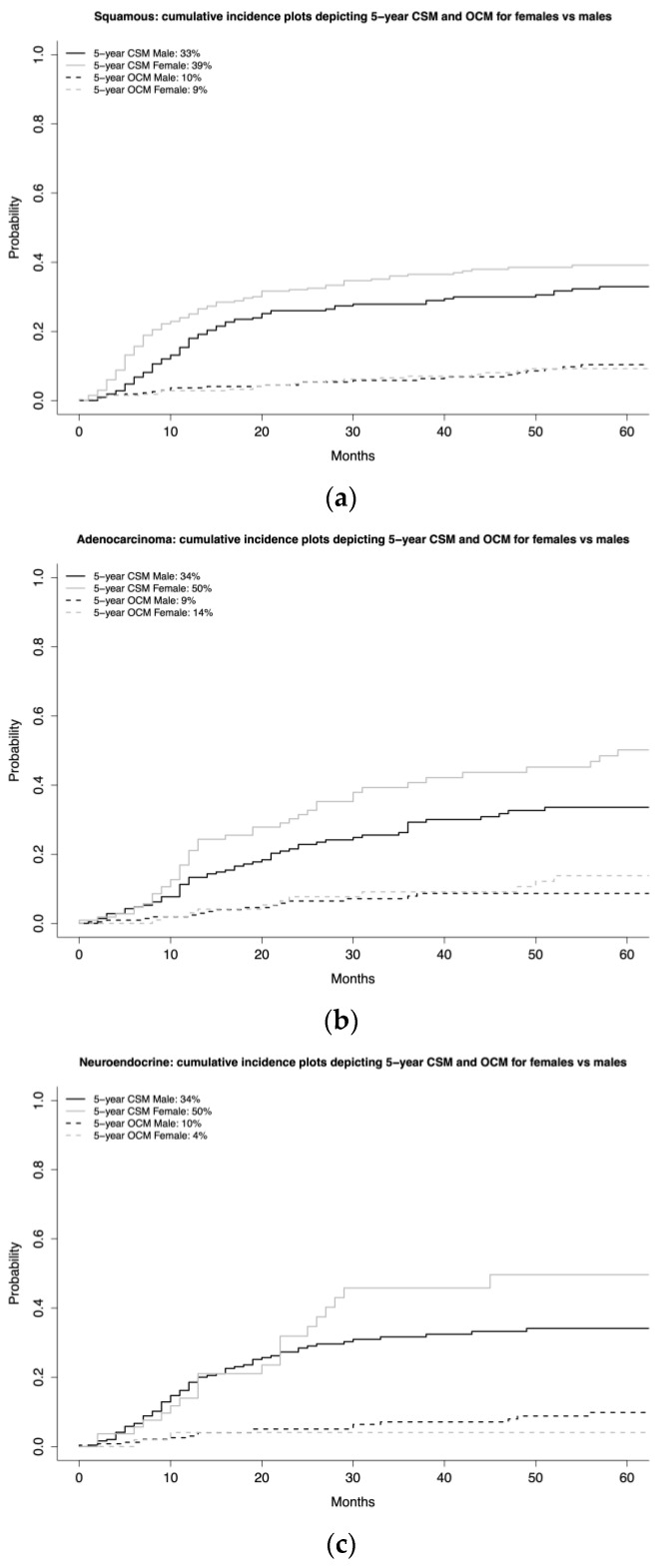

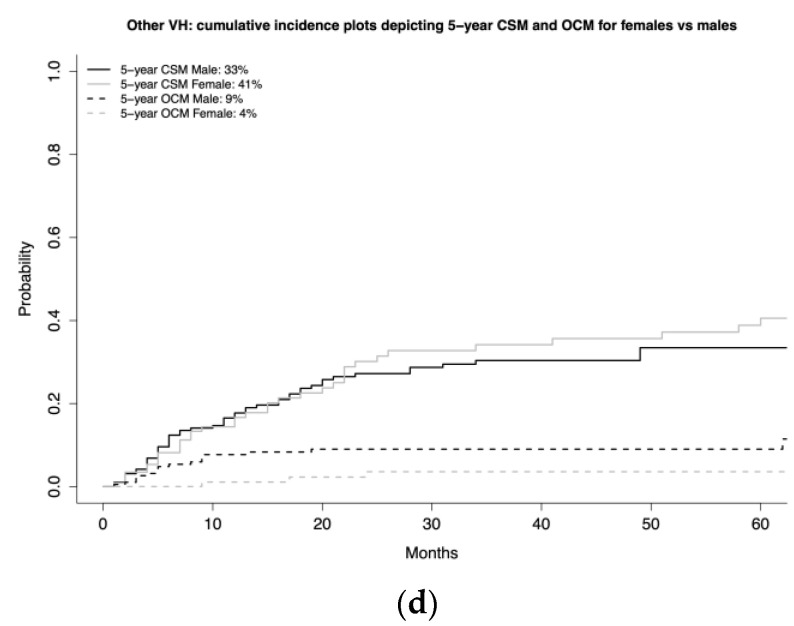
Cumulative incidence plots depicting 5-year cancer-specific mortality (CSM) and 5-year other-cause mortality (OCM), according to sex (female vs. male) in squamous cell carcinoma (SCC) (**a**), adenocarcinoma (ADK) (**b**), neuroendocrine tumor (NE) (**c**), and other VH subgroups (**d**).

In the SCC subgroup (*n* = 671), five-year CSM rates were 39% for females vs. 33% for males (Figure 4). This translated into a CRR HR of 1.33 (95% CI 1.01–1.75, *p* = 0.045), after adjustment for age, T-stage, N-stage, age, and OCM (Table 2).

In the ADK subgroup (*n* = 331), five-year CSM rates were 50% for females vs. 34% for males (Figure 4). This translated into a CRR HR of 1.39 (95% CI 0.94–2.06, *p* = 0.1) after adjustment for age, T-stage, N-stage, and OCM (Table 2). In the NE subgroup (*n* = 304), five-year CSM rates were 50% for females vs. 34% for males (Figure 4). This translated into a CRR HR of 1.24 (95% CI 0.77–2.00, *p* = 0.4) after adjustment for age, T-stage, N-stage, CHT, and OCM (Table 2). In the other VH subgroup (*n* = 317), five-year CSM rates were 41% for females vs. 33% for 142 males (Figure 4). This translated into a CRR HR of 1.06 (95% CI 0.69–1.63, *p* = 0.8) after adjustment for age, T-stage, N-stage, and OCM (Table 2).

## 4. Discussion

We hypothesized that female sex is associated with both pathologic stage and CSM in patients with VH BCa treated with comprehensive RC. Moreover, we tested whether CSM sex-related differences also apply to stage-specific and/or VH-specific subgroups. We tested these three hypotheses and observed important sex-related differences.

First, we identified 1623 patients with VH BCa treated with RC. Of those, 38% were females. Females were a minority in ADK (33%) and NE (18%), as well as other VHs (37%) but not in SCC (51%). These observations, except for SCC, agree with findings on UCUB treated with comprehensive RC, where Rosiello et al. reported a 23% female sex prevalence (SEER 2004–2016) [8]. Interestingly, recurrent urinary tract infections (UTIs) are a risk factor for SCC [14], and females are at increased risk of recurrent UTIs in comparison with males. Consequently, the prevalence of recurrent UTIs among females may represent an explanation for the high rate of SCC. We also observed sex-specific stage distribution differences. Specifically, females had NOC VH BCa more frequently than males when all VH patients were assessed (68% vs. 58%), as well as in SCC (73 vs. 66%) and ADK (71 vs. 59%) subgroups, but not in NE (62 vs. 54%) and other VH (52% vs. 47%) subgroups. Moreover, female sex was an independent predictor of NOC in the overall VH BCa cohort (OR 1.55, *p* = 0.0001), as well as in SCC (OR 1.40, *p* = 0.047) and ADK (OR 1.66, *p* = 0.044) subgroups, but not in NE (OR 1.38, *p* = 0.3) and other VH (OR 1.29, *p* = 0.3). Taken together, females represent a minority in VH BCa, except for the SCC subgroup where their proportion is virtually equally to that of males. However, females had NOC VH BCa more frequently than their male counterparts.

Second, we examined the association between female sex and CSM. Here, we accounted for other-cause mortality using CRR models to provide the most unbiased estimate of CSM [15]. In the overall VH BCa cohort, female sex was associated with higher CSM (HR 1.25, *p* = 0.02). In stage-specific subgroup analyses (NOC vs. OC), female sex was an independent predictor of higher CSM in OC (HR 1.65, *p* = 0.02), but not in NOC (HR 1.17, *p* = 0.1) subgroups. Finally, in VH-specific subgroup analyses, females exhibited higher five-year CSM rates in SCC (39 vs. 33%), ADK (50 vs. 34%), and NE (50 vs. 34%) subgroups, but not in the other VH (41% vs. 33%) subgroup. These differences translated into a statistically significant CRR HR for females vs. males only in SCC (1.33, *p* = 0.045). Moreover, a clinically meaningful CRR HR was also recorded in ADK (1.39, *p* = 0.1) and NE subgroups (1.24, *p* = 0.4), although both lacked statistical significance. Finally, neither statistically significant nor clinically meaningful differences in CSM were observed in the other VH subgroup (1.06, *p* = 0.8). These observations indicate that the female sex disadvantage is mostly operational in the OC and SCC subgroups. Moreover, female sex is also disadvantageous with respect to CSM in ADK and NE. However, the small sample size in these two subgroups potentially undermined the statistical significance of these results. The rarity of VH BCa and the even greater rarity of VH BCa-specific subgroups represents a major limiting factor in this part of the analysis.

Stage disadvantage in females may reflect a female-specific delay in BCa diagnosis. Indeed, sex-related differences in referral patterns were reported by Mansson et al. [16]. Specifically, a higher proportion of females vs. males with BCa were referred to a department other than urology (26.9% vs. 3.7%) for an initial hematuria work-up. Similarly, Ark et al. observed that females were less likely than males to be referred to a urologist (OR 0.59) [17]. Moreover, Cohn et al. reported significantly longer delays from initial hematuria diagnosis to urological assessment in females vs. males (85.4 vs. 73.6 days, *p* < 0.001) [18]. These findings are not surprising, since in females, most general practitioners associate microscopic or even gross hematuria with urinary tract infections [19]. Since delay in diagnosis tends to be greater in females than males, it may be postulated that such delay introduces a stage disadvantage in females, as was observed in the current study. These observations are consistent with previous findings regarding UCUB. Consequently, efforts should be made to maximally reduce or even eliminate diagnostic delays in all females patients at risk of BCa, including VH BCa [20,21]. The female sex disadvantage extends beyond stage at diagnosis. Specifically, female sex is associated with higher CSM after comprehensive RC. The current results validated this hypothesis in the overall VH BCa cohort, including highly statistically significant multivariable CRR HR, even after adjustment for OCM. Similarly, in the stage-specific subgroup analyses, a female-specific CSM disadvantage was observed in OC but not in NOC VH BCa after multivariable adjustments, even for OCM. Finally, in VH-specific subgroup analyses, female sex was associated with a higher CSM in SCC, as evidenced by statistically significant multivariable CRR HR, even after adjustment for OCM. Moreover, in the ADK and NE subgroups, female sex was associated with clinically meaningful higher CSM. However, the small sample size of these VH-specific subgroups undermines the statistical significance of these comparisons. Taken together, female sex clearly predisposes to worse CSM in the overall BCa cohort VH, and this effect is clearly detectable in SCC, ADK, and NE, but not in other VH subgroups.

It is important to emphasize the combined detrimental effect of female sex in both stage distribution (NOC) and CSM in VH BCa. Specifically, in the entire VH BCa cohort, as well as in the SCC subgroup, a highly statistically significant association between female sex and both NOC stage and CSM was observed. In consequence, it may be postulated that in VH BCa, female patients not only present with higher stage but independently of stage, female sex is also disadvantageous with respect to survival. This observation is consistent with the same two-step female disadvantage in UCUB reported by Rosiello et al. [8], where female sex is associated with a more advanced stage at presentation, and fully independently of stage, female sex is also associated with a higher CSM.

To the best of our knowledge, we are the first to report this association in patients with VH BC treated with comprehensive RC. Despite the novelty of our findings, our observations are limited in several regards. First, our findings are based on limited sample size, especially within VH-specific subgroup. However, the current cohort represents the largest group of patients with VH BCa treated with RC. Second, the SEER database does not include detailed preoperative information regarding (1) the time interval from diagnosis to RC, (2) the number of recurrences, (3) the use of intravesical therapy, (4) the number and extent of previous trans-urethral bladder tumor resection (TURBT), (5) TURBT completeness, (6) tumor multifocality, (7) tumor size, (8) presence of associated CIS, and (9) molecular or mutational characteristics, as well as other similar pathological variables that may have been systematically worse in women [22,23,24,25,26]. Third, a centralized pathology review is not available within the SEER database. Fourth, SEER database findings are applicable only to patients from the United States and are not generalizable to other healthcare settings. These, as well as all other limitations related to the retrospective, population-based nature of the SEER database, apply to this research and to other similar analyses that were based on other similar large-scale data repositories (National Cancer Data Base, National Inpatient Sample, SEER-Medicare, or National Surgical Quality Improvement Program).

However, no prospective studies investigating the role of sex in VH BCa treated with RC have been published so far. Consequently, the current findings represent the strongest, more robust, and most generalizable proof of the association of female sex with the outcome of VH BCa treated with RC.

## 5. Conclusions

In VH BC patients treated with comprehensive RC, female sex is associated with a more advanced stage. Fully independently of stage, female sex is also associated with a higher CSM.

## Figures and Tables

**Table 1:** Descriptive characteristics of 1623 VH BCa treated with RC patients, according to sex (female vs. male).

Characteristic	*n*	Overall *n* = 1623	Females *n* = 621 (38%)	Males *n* = 1002 (62%)	*p*-Value ^2^
**Age**	1623	67 (58–75) ^1^	67 (57–75) ^1^	68 (59–75) ^1^	0.084
**Histological Variants ^3^**	1623				<0.001
Squamous		671 (41%)	340 (51%)	331 (49%)	
Adenocarcinoma		331 (20%)	109 (33%)	222 (67%)	
Neuroendocrine		304 (19%)	55 (18%)	249 (82%)	
Other		317 (20%)	117 (37%)	200 (63%)	
**T-stage**	1623				0.001
Ta/Tis		51 (3.1%)	17 (2.7%)	34 (3.4%)	
T1		133 (8.2%)	44 (7.1%)	89 (8.9%)	
T2		492 (30.3%)	159 (25.6%)	333 (33.2%)	
T3-T4		947 (59%)	401 (64%)	546(54%)	
**N-stage**	1623				0.3
N+		407 (25%)	165 (27%)	242 (24%)	
**Stage**	1623				<0.001
Non-organ-confined		100 (62%)	421 (68%)	579 (58%)	
**Perioperative Chemotherapy**	1623				<0.001
Yes		554 (34.1%)	176 (28.3%)	378 (37.7%)	

^1^ Median (IQR), ^2^ Wilcoxon rank sum test; Pearson’s Chi-squared test, ^3^ Row proportions are reported for each VH according to sex (female vs. male).

**Table 2:** Multivariable logistic regression models predicting non-organ-confined (NOC) stage and multivariable competing risks regression models predicting cancer-specific mortality (CSM) according to sex (female vs. male) in the overall cohort of non-urothelial variant-histology bladder cancer (VH BCa) and within VH-specific subgroup: squamous cell carcinoma (SCC), adenocarcinoma (ADK), neuroendocrine tumor (NE), and other VHs.

	Multivariable Logistic Regression (Non-Organ- Confined, NOC)			Multivariable Competing Risks Regression (Cancer-Specific Mortality, CSM)		
		OR (95%CI)	*p*-Value		HR (95%CI)	*p*-Value
Overall cohort	(*n* = 1623) Females	1.55 (1.26–1.92)	0.0001	(*n* = 1623) Females	1.25 (1.04–1.50)	0.02
Stage-specific subgroup analyses				OC (*n* = 623) Females	1.65 (1.08–2.52)	0.02
				NOC (*n* = 1000) Females	1.17 (0.96–1.43)	0.1
VH-specific subgroup analyses	SCC (*n* = 671) Females	1.40 (1.01–1.95)	0.047	SCC (*n* = 671) Females	1.33 (1.01–1.75)	0.045
	ADK (*n* = 331) Females	1.66 (1.02–2.74)	0.044	ADK (*n* = 331) Females	1.39 (0.94–2.06)	0.1
	NE (*n* = 304) Females	1.38 (0.76–2.55)	0.3	NE (*n* = 304) Females	1.24 (0.77–2.00)	0.4
	Other VH (*n* = 317) Females	1.29 (0.81–2.06)	0.3	Other VH (*n* = 317) Females	1.06 (0.69–1.63)	0.8

## Data Availability

The data presented in this study are openly available at https://seer.cancer.gov/.
